# Design of a randomized controlled trial to Link Infectious and Narcology Care (LINC-II) in St. Petersburg, Russia

**DOI:** 10.1186/s13722-020-0179-8

**Published:** 2020-01-13

**Authors:** Natalia Gnatienko, Dmitry Lioznov, Anita Raj, Elena Blokhina, Sydney Rosen, Debbie M. Cheng, Karsten Lunze, Sally Bendiks, Ve Truong, Natalia Bushara, Olga Toussova, Emily Quinn, Evgeny Krupitsky, Jeffrey H. Samet

**Affiliations:** 1 Clinical Addiction Research and Education (CARE) Unit, Section of General Internal Medicine, Department of Medicine, Boston Medical Center, 801 Massachusetts Avenue, 2nd Floor, Boston, MA 02118 USA; 2 Pavlov University, L’va Tolstogo St. 6-8, St. Petersburg, 197022 Russian Federation; 3 Research Institute of Influenza, 15/17, Prof. Popov Street, St. Petersburg, 197376 Russian Federation; 4 Center On Gender Equity and Health, Department of Medicine, University of California San Diego, La Jolla, CA 92093 USA; 5 Department of Education Studies, Division of Social Sciences, University of California San Diego, La Jolla, CA 92093 USA; 6 Department of Global Health, Boston University School of Public Health, 801 Massachusetts Avenue, 3rd Floor, Boston, MA 02118 USA; 7 Health Economics and Epidemiology Research Office, Department of Internal Medicine, School of Clinical Medicine, Faculty of Health Sciences, University of the Witwatersrand, Johannesburg, South Africa; 8 Department of Biostatistics, Boston University School of Public Health, 801 Massachusetts Avenue, 3rd Floor, Boston, MA 02118 USA; 9 Clinical Addiction Research and Education (CARE) Unit, Section of General Internal Medicine, Department of Medicine, Boston University School of Medicine/Boston Medical Center, 801 Massachusetts Avenue, 2nd Floor, Boston, MA 02118 USA; 10 Biostatistics and Epidemiology Data Analytics Center, Boston University School of Public Health, 85 East Newton Street, M921, Boston, MA 02118 USA; 11 St. Petersburg Bekhterev Research Psychoneurological Institute, Bekhtereva St., 3, St. Petersburg, 192019 Russian Federation; 12 Department of Community Health Sciences, Boston University School of Public Health, 801 Massachusetts Avenue, 2nd Floor, Boston, MA 02118 USA

**Keywords:** HIV, PWID, Case management, Rapid ART, Naltrexone

## Abstract

**Background:**

If Russia is to achieve the UNAIDS 90-90-90 HIV targets, better approaches to engage, effectively treat, and retain patients in care are needed. This paper describes the protocol of a randomized controlled trial (RCT) testing the effectiveness of LINC-II, a strength-based case management program for HIV-positive people who inject drugs (PWID) to increase rates of HIV viral suppression, ART initiation, and opioid abstinence.

**Methods:**

This RCT will enroll and randomize 240 participants, recruited from a narcology (addiction care) hospital in St. Petersburg, Russia. Participants are randomized to the intervention or control arms. Those in the intervention arm receive: (1) strengths-based HIV case management supporting coordinated care; (2) rapid ART initiation; and (3) pharmacotherapy for opioid use disorder. We will evaluate the intervention’s effectiveness compared to standard of care on the following outcomes: (1) undetectable HIV viral load at 12 months (primary); (2) initiation of ART within 28 days of randomization; (3) change in CD4 count from baseline to 12 months; (4) retention in HIV care (i.e., ≥ 1 visit to medical care in 2 consecutive 6 month periods); (5) undetectable HIV viral load at 6 months; and (6) past 30-day opioid abstinence (at 6 and at 12 months).

**Discussion:**

This RCT will assess the LINC-II intervention in an urban Russian setting. If effective, it will offer a new approach for increasing the uptake of both HIV and opioid use disorder treatment and coordination of these modalities in standard Eastern European clinical settings.

*Trial registration* This study was registered with ClinicalTrials.gov through the National Institutes of Health, NCT03290391. Registered 19 September 2017, https://clinicaltrials.gov/ct2/show/NCT03290391

## Background

With a growing HIV epidemic in Russia driven in part by injection drug use (IDU), it is critical to develop approaches for improving the cascade of HIV care for people who inject drugs (PWID) [1]. Effective approaches will need to address engagement (i.e., initiation and retention) in HIV care and receipt of effective treatment, as only an estimated 10% of HIV-positive PWID in St. Petersburg are currently receiving antiretroviral therapy (ART) [2]. For Russia to make progress toward the UNAIDS 90-90-90 targets [i.e., 90% aware of HIV diagnosis, 90% of those diagnosed on antiretroviral therapy (ART), and 90% of those on ART with suppressed HIV viral load (HVL)], a bold new strategy is required. Our prior study, Linking Infectious and Narcology Care (LINC), in St. Petersburg documented that strengths-based case management initiated in Russia’s narcology (addiction care) treatment system and delivered in five sessions over 6 months significantly increased HIV-positive PWID’s linkage to HIV care and receipt of appropriate HIV care [i.e., prescribed ART or having a second CD4 count if CD4 > 350 (the threshold for ART initiation at the time of the study) within 12 months of enrollment] [3]. Increases of these care outcomes were modest, and significant effects of the LINC intervention on retention in care and CD4 count were not observed. Reasons for this, identified through qualitative research, included complex pathways to access ART, which required up to eight visits to a stand-alone HIV treatment facility; active substance use; stigma related to both addiction and HIV; and an inadequate duration of the case management intervention [4, 5]. Better approaches to engage, effectively treat, and retain patients in care are clearly needed. Building on our previous work in Russia, we are now evaluating a new pragmatic intervention for HIV-positive PWID combining and coordinating three strategies: strengths-based case management, pharmacotherapy for opioid use disorder, and rapid initiation of ART.

## Methods/design

Linking Infectious and Narcology Care—Part II (LINC-II) is a randomized controlled trial (RCT) enrolling 240 HIV-positive PWID to test the effectiveness of the LINC-II intervention. We will also evaluate the impact of coordinating care using qualitative methods and surveys and will assess the intervention’s costs and cost-effectiveness. Eligible participants are randomly assigned to either the LINC-II intervention or the control group (narcology hospital’s standard of care). The intervention involves 12 months of strengths-based case management, rapid initiation of ART, and 12 months of pharmacotherapy for opioid use disorder (OUD).

We will evaluate the effectiveness of the intervention compared to standard of care on the following outcomes: (1) undetectable HIV viral load at 12 months (primary); (2) initiation of ART within 28 days of randomization; (3) change in CD4 count from baseline to 12 months; (4) retention in HIV care (i.e., ≥ 1 visit to medical care in 2 consecutive 6 month periods); (5) undetectable HIV viral load at 6 months; and (6) past 30-day opioid abstinence (at 6 and at 12 months).

### Study setting

**Fig. 1:**
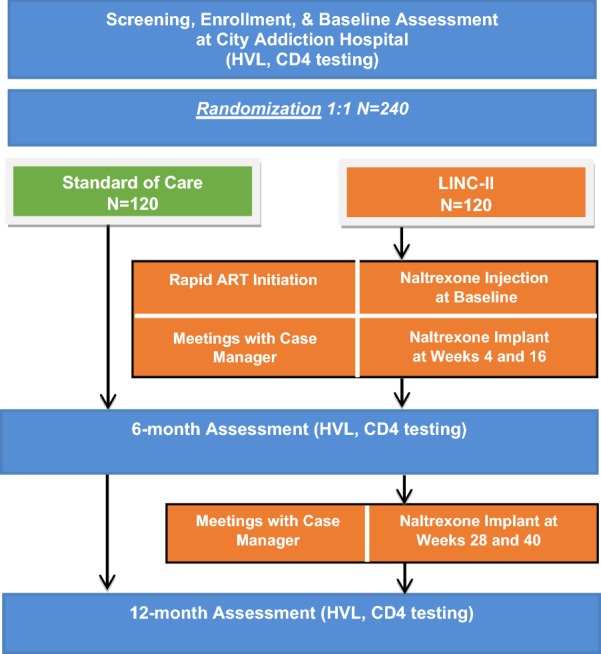
LINC-II study design

The study is being implemented at three locations in St. Petersburg (see Fig. 1 for study flow). St. Petersburg’s City Addiction Hospital is the site of participant recruitment. City Addiction Hospital is a government-funded institution that provides free addiction care services to St. Petersburg residents who are registered as having a drug or alcohol use disorder. Services include detoxification, early stabilization, and inpatient rehabilitation and are provided free of charge. The typical length of stay for hospitalized patients is 1 to 4 weeks. St. Petersburg City AIDS Center provides HIV care for St. Petersburg citizens, including LINC-II study participants. A typical initial outpatient visit to initiate HIV care includes examination by an infectionist (physician), lab testing (e.g., HVL, CD4 count), and several referrals for mandatory attestations to initiate ART (e.g., TB specialist, psychologist or addiction specialist, ophthalmologist, etc.). St. Petersburg City AIDS Center has a staff narcologist to refer HIV-positive PWID to addiction treatment clinics. HIV case managers (CMs), including some HIV-positive PWID in recovery (i.e., peers) have been introduced at the HIV clinics. ART is provided at no cost to all eligible patients in Russia. Follow-up research study visits and adverse event monitoring occur at the Laboratory of Clinical Pharmacology of Addictions at the First St. Petersburg Pavlov State Medical University in St. Petersburg, Russia. This university is the major educational, scientific, and clinical medical institution for northwestern Russia.

### Participants and recruitment

Study eligibility criteria are listed in Table 1. Exclusion criteria were primarily based on the need for intramuscular injections and contraindications for naltrexone use. Recruitment takes place at two or more departments of the St. Petersburg City Addiction Hospital. Patients are typically screened for study eligibility 3 to 5 days after admission to the department participating in LINC-II at the narcology hospital. Screening for the LINC-II study takes place in two steps: (1) a pre-screen conducted through chart review; and (2) in-person screening of participants who are eligible following the pre-screen. Pre-screening is conducted by narcologists (Addiction Psychiatrists) within the department. Research Assessors (RAs) who are City Addiction Hospital staff (psychologists not involved in the patient’s care) conduct the in-person screening. If a patient is identified by the narcologist as meeting criteria based on chart review, the narcologist notifies the RA that a patient in their department is available to be screened for the study. The RA is introduced to the patient by the narcologist and meets with the patient in a private location (e.g., hospital room or exam room) to provide a description of the study and conduct in-person screening to assess whether the patient meets eligibility criteria for the study.

**Table 1:** LINC-II eligibility criteria

Inclusion criteria	Exclusion criteria
Age 18 years or older	Not fluent in Russian
HIV-positive	Cognitive impairment precluding informed consent
Hospitalized at narcology hospital	Breastfeeding or being pregnant
History of injection drug use	ART use in the past 30 days prior to hospitalization
Provision of two contacts to assist with follow-up	Acute severe psychiatric illness (i.e., answered yes to any of the following: past three month active hallucinations; mental health symptoms prompting a visit to the emergency room or hospital; mental health medication changes due to worsening symptoms; presence of suicidal ideations)
Address within St. Petersburg or districts within 100 km of St. Petersburg	Known history of liver failure
Possession of a home or mobile phone	Known hypersensitivity to naltrexone
Ability and willingness to comply with all study protocols and procedures	Aspartate aminotransferase (AST) or alanine aminotransferase (ALT) greater than 5 times the upper limit of normal
	Severe thrombocytopenia
	Known coagulation disorder/taking anticoagulation medications
	Body habitus that precludes intramuscular injection (e.g., BMI < 17 or > 45)
	Known history of Raynaud’s disease, Itsenko-Cushing syndrome, generalized mycoses, glaucoma, or osteoporosis
	Planned surgeries in the next 12 months

After confirming study eligibility, the RA administers and documents informed consent, and enrolls the patient into the study. The RA then collects contact information for the participant and at least two alternative contacts before conducting the baseline assessment, followed by randomization.

### Randomization

Randomization is stratified based on one factor that is expected to predict study outcomes: ever ART use. Blocked randomization using random block sizes of two and four are used within each stratum. A computer-generated randomization table was created to allow randomization to occur electronically via REDCap.

### Intervention

The LINC-II intervention has three main components: (1) strengths-based HIV case management; (2) rapid ART initiation; and (3) pharmacotherapy for opioid use disorder.

#### Strengths-based case management

As part of the strengths-based case management intervention, a peer (i.e., man or woman living with HIV and in stable recovery from addiction) case manager works one-on-one with patients to support and motivate their client in accessing HIV care, reducing barriers to HIV care, and maintaining their addiction treatment. Strengths-based case management rests on the premise that all patients have strengths, and, once recognized, these strengths can be the foundation for them to build their capacities to engage in HIV medical care and improve their HIV-related health outcomes [6]. The strengths-based approach is based on Social Cognitive Theory, emphasizing the client’s self-efficacy to create change, and includes psychoeducational support and counseling, which are aimed at helping patients gain additional knowledge about HIV care and ways to independently access available services. This approach also utilizes components from the Psychological Empowerment Theory, mainly in the way patients are supported by CMs in identifying their own strengths to improve healthcare-seeking behavior [7, 8].

The CM serves as a coordinator between the narcology and HIV systems of care, delivering HIV strengths-based case management via ten one-on-one sessions over a 12-month period. The first LINC-II case management session is preferably held at the City Addiction Hospital within days of study enrollment; and the second session is scheduled to take place at the City AIDS Center. Sessions are planned to occur at approximately 3 to 6-week intervals, with a goal of five sessions for every 6 months of intervention, for a total of ten sessions. Following session two, subsequent case management encounters can occur at any location of preference and via phone, if necessary.

The first two sessions consist of determining the patient’s psychological and resource-related strengths and developing goals related to HIV care and supporting recovery. CMs discuss with participant their most recent CD4 count and HVL results, if they are available, and the benefits of regular HIV care. Follow-up sessions reinforce prior sessions, reviewing previously set goals and patients’ strengths, creating new goals as needed.

LINC-II offers SMS messaging between sessions to reinforce the CM-patient relationship and contact. SMS are sent to participants, encouraging them to contact their CM if they so wish in times of need (e.g., a craving episode). CMs can also choose to replace the SMS with phone calls, if that mode of communication is preferred by the participant.

#### Rapid access to ART

The World Health Organization’s guidelines for HIV treatment call for “rapid” initiation of ART, defined as starting treatment within 7 days of diagnosis [9]. With all HIV-positive individuals now eligible for ART in Russia, regardless of CD4 count, participants randomized to the intervention group are offered initiation of ART as quickly as possible, ideally while at the City Addiction Hospital. Rapid access to ART is facilitated by the City AIDS Center’s infectionist (HIV physician), who sees patients at the St. Petersburg City Addiction Hospital. All participants meet with the infectionist while hospitalized and have their blood drawn for CD4 count and HVL testing. As customary in Russia, all ART initiation requests in St. Petersburg must be approved by a special committee at the City AIDS Center. For participants randomized to the intervention group, the infectionist facilitates this approval with the ultimate goal of starting participants on ART while they are still hospitalized at the City Addiction Hospital. If a participant remains hospitalized at the time of ART approval, the infectionist delivers the first set of medications, a 1-month supply, to the patient while in the hospital. The CM helps schedule the subsequent visit to the City AIDS Center 1 month later to pick up the next refill of medication. If a participant is no longer at the City Addiction Hospital, then the CM will help the patient schedule his or her visit to the City AIDS Center as soon as possible, where the first set of medications will be provided. Once the initial visit to the City AIDS Center is made, participants continue HIV care at one of two possible sites: City AIDS Center or their local HIV outpatient clinic; all subsequent ART refills can be picked up at a local clinic.

#### Pharmacotherapy for opioid use disorder

Opioid agonist treatment (i.e., buprenorphine and methadone) is not available in Russia. Participants in the intervention arm receive one intramuscular gluteal injection of 380 mg of naltrexone for extended-release injectable suspension at the City Addiction Hospital. Since naltrexone can precipitate withdrawal in participants with a physiological dependence on opioids, participants are given a naloxone challenge prior to receiving naltrexone. Following hospital discharge, participants come to First St. Petersburg Pavlov State Medical University to receive a 1000 mg dissolvable naltrexone implant. Participants receive 4 naltrexone implants while in the study—every 10 to 12 weeks starting at week four post-study enrollment (weeks 4, 16, 28, 40), unless the participant relapses. Participants undergo a naloxone challenge at study visits during which the naltrexone implant is inserted and only if the participant’s urine is opioid-free. If participant does not pass the naloxone challenge due to relapse, they are referred to detox treatment and are invited to return upon completion of that treatment for a repeat naloxone challenge. Naltrexone implantation is an outpatient procedure conducted on site by a surgeon with the assistance of a study nurse. Participants are invited to return seven to 11 days following the implant for removal of sutures and every 4 weeks following the implant for a medication check-in visit. Assessors administer brief counseling (~ 5 min) to participants during all medication visits.

### Control

Participants in the control group receive standard care as normally provided to patients in the narcology hospital. This could include detoxification with medications, substance use counseling, and treatment for comorbid psychiatric conditions, as well as possible inpatient rehabilitation for up to 30 days. Detoxification typically takes 5–7 days and the most commonly used medications are clonidine, antidepressants, non-opioid analgesics, hypnotics, and loperamide. Stabilization occurs in the same department within an additional 1–2 weeks with additional similar care. Patients also meet with an infectionist and have their blood drawn and tested for CD4 count and HVL while at the hospital. Upon narcology hospital admission, HIV testing is routinely performed on all patients who are not documented to be HIV-positive. Prior to discharge, those identified as HIV-positive are given contact details for the AIDS Center, but not an appointment. Upon discharge, patients are recommended to receive outpatient narcology treatment monthly, for 1 year. Participants who visit the City AIDS Center are able to receive ART medication, provided they undergo required pre-ART testing and examinations, including a purified protein derivative (PPD) skin test; and referrals to TB specialists, addiction psychiatrists, psychologists, etc., which takes about 1 month. Enrollment in the study does not preclude participants from receiving any HIV or narcology care that would normally be accessible to them. Participants in both study arms are also given printed information, including phone numbers, on places that provide HIV medical care and are referred to outpatient narcology care.

### Assessments

Participants are assessed at baseline, 6, and 12 months post enrollment, along with shorter medication visits for the intervention group occurring every 4 weeks. All follow-up study assessments take place at First St. Petersburg Pavlov State Medical University. Participants are compensated for their time and travel with 1000 rubles (approximately 15 USD) at baseline, 500 rubles (approximately 8 USD) at medication visits, 1500 rubles (approximately 23 USD) at medication visits in which implantable naltrexone is inserted, and 2000 rubles (approximately 30 USD) at 6 and 12-month follow-up visits. We will review medical charts to obtain City AIDS Center HIV treatment history, including information on secondary study outcomes of initiation of ART and retention in HIV care.

#### Questionnaires

The components of baseline and follow-up assessments are listed in Table 2. Most sections of the study questionnaire are interviewer-administered with sections deemed to contain potentially sensitive questions self-administered by the participant. In addition to study questionnaires, at each study visit RAs measure and record participants’ weight and blood pressure.

**Table 2:** Assessment table of participants in LINC-II study (n = 240)

Administered assessment	Study time point		
	Baseline	6-month	12-month
Demographics [24–26]	X		X
HIV testing and HCV diagnosis [27]	X		X
ART use and adherence [28]	X	X	X
Health care utilization [29]	X	X	X
Costs of illness and treatment in last month	X	X	X
Attitudes toward care coordination and care continuity [30]	X	X	X
Barriers to medical care [31]	X	X	X
Perceived discrimination in health care	X	X	X
DSM-5 opioid use disorder	X		
Drug use [32, 33]	X	X	X
TLFB: opioids [34, 35]	X	X	X
Tobacco use [36, 37]	X		
Alcohol use: AUDIT [38]	X		X
Opportunistic infections [39]		X	X
Medications	X	X	X
Pain assessment [40, 41]		X	X
HIV sex risk behaviors and reproductive health [42, 43]			X
Sexual partners^a^	X		X
HIV risk categories^a^ [44, 45]	X		
HIV disclosure^a^ [46, 47]	X		
HIV stigma^a^ [48]	X		X
Substance use stigma^a^	X		X
Barriers to medical care part 2^a^ [31]	X	X	X
Depressive symptoms (CES-D)^a^ [49, 50]	X		X
Anxiety (GAD-7)^a^ [51]	X		X
Case manager questions^a^		X	X
Partner violence and sexual assault^a^ [52]		X	
Overdose and suicide^a^		X	X
Social support scale [53]	X		
VR-12 health survey—MOS-HIV [54, 55]	X		X
Primary activity	X	X	X
Visit costs		X	X

^a^Self-administered

#### Laboratory testing and results

This study requires the collection of blood at baseline, 6, and 12 months. As a precaution, liver toxicity [aspartate aminotransferase/alanine aminotransferase (ALT/AST)] testing occurs for intervention participants at implant visits two, three, and four, and if either liver enzyme is greater than five times the upper limit of normal, results are communicated to the participant during their suture removal visit and a repeat test is conducted during their next medication visit. If any repeat test result is again greater than five times the upper limit of normal, or if a patient is symptomatic (e.g. fatigue, anorexia, jaundice, nausea, vomiting, dark urine, light stool, abnormal pain), the patient is referred to a hepatologist for further evaluation and a recommendation about continuing study medication. The samples are tested for CD4 count and HVL at baseline, 6, and 12 months at the City AIDS Center, and only if participants do not already have a recent result in their medical record.

### Adverse events and safety monitoring

For intervention participants, safety is monitored by study staff every 4 weeks during in-person medication visits. Urine pregnancy tests are administered to all women at each visit. Symptoms are assessed at baseline to document any chronic conditions or symptoms that existed prior to study enrollment. At each visit, the RA asks participants about any new symptoms experienced since the last study visit. Any event meeting the criteria for an adverse event (AE), serious adverse event (SAE), or unanticipated problem is properly documented and reported. The research team reviews the results of all blood work conducted on participants. Any clinically significant abnormal lab results are recorded as an AE and/or SAE and the participant is referred for care to their medical provider.

### Mixed methods assessment of LINC-II impact on coordinating care

In addition to assessing its effectiveness, the study will evaluate the intervention’s impact on coordinating care across the narcology and HIV health care systems, using mixed methods data from health care providers and administrators in both the HIV and narcology systems, as well as from LINC-II study participants. Survey data and interviews will assess perceptions of whether the narcology and HIV care systems increased coordination of care over the period of the study and, qualitatively, how and why coordination does or does not occur.

We will recruit 50 providers and administrators from HIV and narcology care systems to participate in a survey at study launch and up to 26 providers to participate in in-depth interviews and short surveys at study launch as well as 12 and 24-months post-launch.

All study participants complete a brief survey assessing their experiences with coordinated care at 6 and 12-month study visits. All LINC-II participants will also be asked about their perceptions of the intervention as part of the follow-up survey. Additionally, a subset of LINC-II participants at each time point (n = 10:5 intervention, 5 control) will be invited to participate in qualitative interviews to discuss their experiences with care coordination.

### Cost and cost-effectiveness estimates

The study will also evaluate whether LINC-II is an affordable and cost-effective strategy for achieving undetectable HVL in HIV-positive PWID. If the intervention is successful, it will be important to understand the net cost to be incurred by the health system and patients and LINC-II’s cost-effectiveness compared to standard of care. This economic perspective will inform policy makers on scaling up the LINC-II approach both within Russia and other countries with HIV epidemics driven by injection drug use.

### Data management

The Boston University Biostatistics & Epidemiology Data Analytics Center (BEDAC) designed, developed, and maintains the electronic data collection (EDC) systems and underlying data structures used for capturing participant, interview, and tracking data. BEDAC implements procedures for data quality control, including built-in skip patterns and range checks for the EDC systems, which serves to minimize errors in data capture and facilitates data transfer to the Boston team in real-time. For instances where the EDC system will serve as the initial data record (e.g. direct data entry of interview data), logic checks are implemented and reviewed with study staff. For instances where data entered in the EDC system are based on source documents (e.g. lab results, paper data collection forms), monthly data audits are conducted to ensure data quality.

### Analytic methods

#### Statistical analyses

Power was calculated for the primary study outcome of undetectable HIV viral load at 12 months post randomization and assumed a two-sided alpha level of 0.05. It was expected that 240 participants would be enrolled into the trial. Based on data from the original LINC study, we expected 10% of controls to have undetectable viral load at 12 months. Given this and assuming 20% loss to follow-up (i.e., 192 evaluable subjects) the study has 80% power to detect an absolute difference of 17% (i.e., 27% vs. 10% in the intervention and control arms, respectively) using a chi-square test with continuity correction. We anticipate larger effects may be observed in our study, which would result in even higher power.

This study will use an intent-to-treat analysis that includes all randomized participants according to their assigned group. Descriptive statistics will be calculated for variables at baseline to assess whether there appear to be any differences across treatment arms.

As the primary study outcome is HIV viral load suppression at 12 months, initial analyses will be performed comparing this binary outcome between groups using a chi-square test. The primary analysis will use multivariable logistic regression analyses to control for the stratification factor, ever ART use, to improve efficiency. If there are any baseline factors that appear to differ by randomized group, additional sensitivity analyses will be conducted controlling for these factors to assess for potential confounding. The secondary outcomes of undetectable viral load at 6 months, ART initiation within 28 days, and retention in HIV care will be analyzed using the same approach described above. Change in CD4 count between baseline and 12 months will be analyzed using multiple linear regression. If the distribution of change in CD4 is skewed, transformations of the data will be considered. A median regression model will be utilized if an appropriate transformation is not identified [10, 11].

We will perform additional analyses to explore potential effect modifiers of the LINC-II intervention. The three potential effect modifiers of interest are: gender, ever ART use, and 30-day IDU. We will fit separate models including two-way interactions between randomization group and each potential effect modifier. If an interaction is significant, subsequent stratified analyses will be conducted to explore and describe the effect of the LINC-II intervention by categories of the moderator.

Exploratory analyses will be conducted to assess potential mechanisms that may drive LINC-II’s ability to improve HIV care outcomes using the Baron and Kenny approach [12]. The three potential mediators we will explore are decreases in substance use, HIV stigma, and substance use stigma. However, because the interpretation of the degree of mediation in logistic models is complicated by their inherent nonlinearity, we will conduct additional analyses using the recently developed causal inference approach to mediation (also referred to as the counterfactual framework), an approach that allows potential interactions between the intervention and mediators and derives direct and indirect effects for binary outcomes [13–15]. We will use the Stata mediation package to conduct these analyses [16, 17].

#### Mixed methods assessment of LINC-II impact on coordinating care

We will conduct descriptive statistics (e.g., means, medians, interquartile ranges, and confidence intervals) to assess perceptions and experiences of coordinated care using quantitative data from provider, administrator, and patient surveys over time. We will also analyze repeated measures of patients’ attitudes and experiences using mixed effects regression models controlling for randomized group to assess overall changes over time and to explore and describe potential differences between study arms. We will analyze qualitative interview data following a thematic approach [18]. Content analysis of qualitative data will reveal themes regarding care coordination and will identify best practices for LINC-II implementation in similar settings, (e.g., where addiction and HIV care systems are largely separate). Qualitative and quantitative results will be triangulated [19].

#### Cost and cost-effectiveness estimates

To estimate cost and cost-effectiveness, we will adapt methods developed by Rosen et al. [20, 21]. When all follow-up to the primary outcome (12-month viral suppression) has been completed, patient resource utilization will be extracted from patients’ medical records for HIV care and from study forms for narcology care in the intervention arm. Narcology care for patients in the control arm will be estimated from patient self-report at the time of 6 and 12-month outcome assessments. Unit costs will be obtained from published information, external suppliers, and the study sites’ finance and procurement records and applied to the resource usage data to provide an average cost per study participant. Costs will be measured from the provider perspective and will include the cost of all resources utilized for each study participant from the date of admission to the narcology hospital for a period of 12 months, including all drugs, laboratory tests, inpatient days, outpatient visits, case manager costs, and fixed costs such as building space, equipment, and administrative staff. For patients referred to local clinics, rather than the study hospital, for ongoing HIV care after ART initiation and/or for narcology care, fixed costs will be estimated at the facility level for a typical local clinic for each type of care.

We will estimate average cost with 95% confidence intervals to the provider per patient enrolled, per patient initiating ART, and per patient achieving viral suppression by 12 months. We will also estimate total cost per patient achieving the primary outcome, which takes into account all the costs for all the patients but divides by only the number with a successful outcome (i.e., 12-month viral suppression) and thereby links the cost of service delivery to the primary outcome. The cost-effectiveness of the intervention, compared to standard care, will also be estimated as an incremental cost per incremental outcome. The cost and cost-effectiveness results will then be evaluated in the context of existing healthcare budgets, resource availability (e.g., trained case managers), other relevant interventions that have been studied in Eastern Europe, and cost estimates for similar countries to help inform policy makers about the affordability and priority of scaling up the program.

In addition to the provider cost estimates described above, the baseline questionnaire will elicit information about patient costs of seeking care, such as transport fares, lost wages, and substitute labor costs (e.g., for childcare). The average cost to participants by arm and by outcome will be estimated and used both to help explain study results (e.g. there may be an association between patient costs and retention in care) and as a component of the overall economic evaluation.

### Protection of study participants and their data

The LINC-II study was approved by the Institutional Review Boards of Boston University Medical Campus and First St. Petersburg Pavlov State Medical University. All study participants complete the informed consent process including written informed consent.

A secure, web-based system is used for data collection. Data transmissions are encrypted using secure socket layering (SSL) and access to the study website and data collection platform is protected via secure logins. Study data and the study website are located on a secure server within the Boston University Medical Campus (BUMC) domain. Participant tracking information is kept separate from research data.

## Discussion and impact

The LINC-II study will assess the effectiveness and implications of an intervention designed to pursue 90-90-90 HIV care cascade targets by facilitating coordination of care between the addiction (i.e., narcology) and HIV treatment systems in Russia. This is high-priority research given that in St. Petersburg, Russia, 50–60% of PWID are HIV-positive, yet among this key population, less than 10% are on ART [2, 22, 23]. Engaging this population in HIV care has immense consequences for the overall HIV epidemic in Russia, as it has the potential to limit onward HIV transmission through successful achievement of undetectable HVL. In order to evaluate the intervention’s potential impact on health care structures and its financial viability, we will assess this strategy’s impact on coordinated care across the narcology and HIV health care systems in Russia, as well as its costs and cost-effectiveness. Results from the study will be most generalizable to HIV-positive individuals hospitalized for narcology care. Potential lessons learned in Russia should be applicable to other countries attempting to engage HIV-positive people in care.

## Data Availability

Data sharing is not applicable to this article as no datasets were generated or analyzed during the current study.

## Abbreviations

AE: adverse event
ALT/AST: aspartate aminotransferase/alanine aminotransferase
ART: antiretroviral therapy
BEDAC: Boston University Biostatistics & Epidemiology Data Analytics Center
BUMC: Boston University Medical Campus
CM: case manager
EDC: electronic data collection
HVL: HIV viral load
IDU: injection drug use
LINC: linking Infectious and Narcology Care
OUD: opioid use disorder
PPD: purified protein derivative
PWID: people who inject drugs
RA: research assessor
RCT: randomized controlled trial
SAE: serious adverse event
SSL: secure socket layering
